# Aspects regarding colour fastness and adsorption studies of a new azo-stilbene dye for acrylic resins

**DOI:** 10.1038/s41598-021-85452-7

**Published:** 2021-03-15

**Authors:** Simona Popa, Maria Elena Radulescu-Grad, Alina Perdivara, Giannin Mosoarca

**Affiliations:** 1 Faculty of Industrial Chemistry and Environmental Engineering, Politehnica University of Timisoara, V. Parvan Bd. No. 6, 300223 Timisoara, Romania; 2 ”Coriolan Dragulescu” Institute of Chemistry, Romanian Academy, Mihai Viteazul Bd. No. 24, 300223 Timisoara, Romania; 3 AZUR S.A, Constructorilor Bd., No. 1-3, 300571 Timisoara, Romania

**Keywords:** Chemistry, Chemical engineering

## Abstract

The aim of this study was the colour fastness investigation of the new synthetized direct symmetrical azo-stilbene dye, using two of the film field domain reference tests, namely the wet-scrub and the UV tests. The dye was incorporated in a water-based resin, and then was applied on a PCV foil. The film colour parameters were determined before and after 200 wet-scrub cycles. Further, the dye was mixed with an acrylo-polyurethanic resin and then was applied on an aluminium plate, which was exposed to UV radiation for 414 h. The film colour parameters were recorded periodically. The maxima of the reflectance spectra depend on the UV time exposure. The quality of the film was analysed by the degree of gloss. A second focus was the elimination of the dye’s traces from wastewaters (these may be resulted from the industries which apply surface coating methods), using the active carbon powder. The main operational adsorption process parameters influence were investigated. Equilibrium, kinetic and thermodynamic studies were performed. The adsorption process was confirmed by the CIEL*a*b* colour space analysis. All colour studies were investigated using UV–Vis spectroscopy.

## Introduction

Azo dyes are by far the most important chemicals, accounting for over 60% of the known commercial dyes^1^. They are widely used in the textiles, leather, cosmetics, and food industries^2–5^. Besides their classical colouring property, azo compounds are known as antibacterial and antioxidant agents^6,7^ and also widespread used in photoelectronics and photonics, applications as detectors for various environmental stimuli, as selective chemosensors for metals detection, etc.^8,9^.

Many industrial surfaces require colour coatings, such are paper, composite, wood, concrete, ceramics, metals (aluminum, or steel) etc. At the same time, in agriculture the coating process is applied to seed, mulch, and other various products. Generally, surface coating method is completed by a thin film that can be functional or decorative^10^.

Organic coatings are classified according to the resin’s chemical composition. The resin is dissolved or suspended in the solvent. Water-based resins show remarkable advantages such are low toxicity, low flammability, and environmental friendly as expected. This category refers to any type of coating formulated based on an acrylic polymer. Acrylic resins have been widely used in various areas: leather, automotive industries, construction fields etc. due to the good stability, gloss and colour retention^11,12^. In contrast to the water-based resins, the solvent-based ones bring performance attributes to architectural coatings, offering durability throughout all applications.

Polyurethane acrylates are materials that can combine high abrasion resistance, breaking strength, low temperature resistance of polyurethanes, with good optical properties, water resistance and weather resistance of acrylates. Their usage in optical coating, printing industries and medicine are well known^13,14^.

The dyes fastness properties are important for their usage in different fields such are: textile, leather, film or print industries. Some of the most known, relevant and applied colour fastness tests are: light, washing, wet and/or dry scrubbing, sublimation, weather etc.^15–22^. The most common colour analysis method is the UV–Vis spectroscopy. The uniform colour space, CIE L*a*b*, was defined for colour communication and is widely adopted today in many industries for colour control and management^23–25^.

The wastewaters from the industries that use dyes in the technological process may contain these chemicals, which cause serious environmental problems, especially related to water pollution: damage on the aesthetic nature of natural effluent, prevention of the sunlight penetration, which disturb photosynthesis and the aquatic equilibrium, ecotoxicity and bioaccumulation potential risk^26–29^.

Various treatment technologies, such are: coagulation, ion exchange, membrane processes, precipitation, electrochemical processes, chemical oxidation, ozonation, adsorption, photocatalytic processes and biodegradation, are available to remove the dyes from water^26,30,37^. Due to the obvious advantages associated with low cost, design simplicity, insensitivity to toxic pollutants, ease of operation and high efficiency, adsorption is the preferred process for the dyes removal from water. Even if its price is relatively high, activated carbon is the most widely used adsorbent due to the high specific surface and the excellent adsorption ability^26,30^.

In this study, our focus was to determine the new dye **I** colour’s fastness under two of the film field domain reference tests, namely the wet-scrub and the UV tests, based on its good colour properties in acrylic resins^31^. Knowing that the wastewaters resulted from the industries which apply surface coating methods may contain traces of dyes, an important part of our work was to eliminate the new dye **I** traces from the wastewater by using a well-known adsorbent material, namely the powdered active carbon (PAC). The effects of the main parameters (contact time, adsorbent dose, initial dye concentration and temperature) that may influence the adsorption process were investigated. Equilibrium and kinetic modelling and thermodynamic study were also conducted. In order to confirm the adsorption results we used the CIE L*a*b* colour space analysis. To the best of our knowledge, this kind of investigation upon the adsorption process was not yet reported.

Taking into account that the dye **I** is a new synthesized symmetrical azo-stilbene dye for acrylic resins, all the studies presented in this paper may be considered as new, original and with possible practical further eco-friendly applications.

## Results and discussion

### Fastness properties

The colour differences between the sample and the standard: ΔE^*^_ab_—the colour difference calculated as a geometric distance between two positions in the CIEL*a*b* space, ΔC^*^—the difference in saturation between the sample and the standard, and Δh^*^—the difference in the hue angle between the sample and the standard, as well as the colour strength (K/S) in the visible region of the reflectance spectrum, may be calculated using the equations presented in the literature^32–34^.

The colour CIEL*a*b* parameters: lightness (L*), redness (a*), yellowness (b*), chroma or saturation (C*) and hue angle (h*) respectively were determined in order to highlight the fastness of the applied new dye **I** incorporated in two types of films, the water-based acrylic resin (dye **I**-WB) and the solvent acrylo-polyurethanic resin (dye **I**-APU). They are mentioned in the Materials and Methods section. The colour of the dye **I**-WB film under the wet-scrub test slightly changed (See Supplementary Information, Fig. S1) according to the colour parameters and to the colour differences (Table 1)^35^.

**Table 1:** The colour properties of the dye **I**-WB at wet-scrub cycles.

Film	dye **I**-WB standard	dye **I**-WB after 200 cycles
L*	70.8 ± 0.2	70.4 ± 0.1
a*	11.2 ± 0.2	15.2 ± 0.2
b*	− 8.6 ± 0.1	− 6.5 ± 0.1
C*	14.1 ± 0.2	16.6 ± 0.1
h*	5.6 ± 0.05	5.9 ± 0.1
ΔE*	–	4.5 ± 0.2
ΔC*	–	2.5 ± 0.2
Δh*	–	3.7 ± 0.1
*K/S*	17.9	17

**Figure 1:**
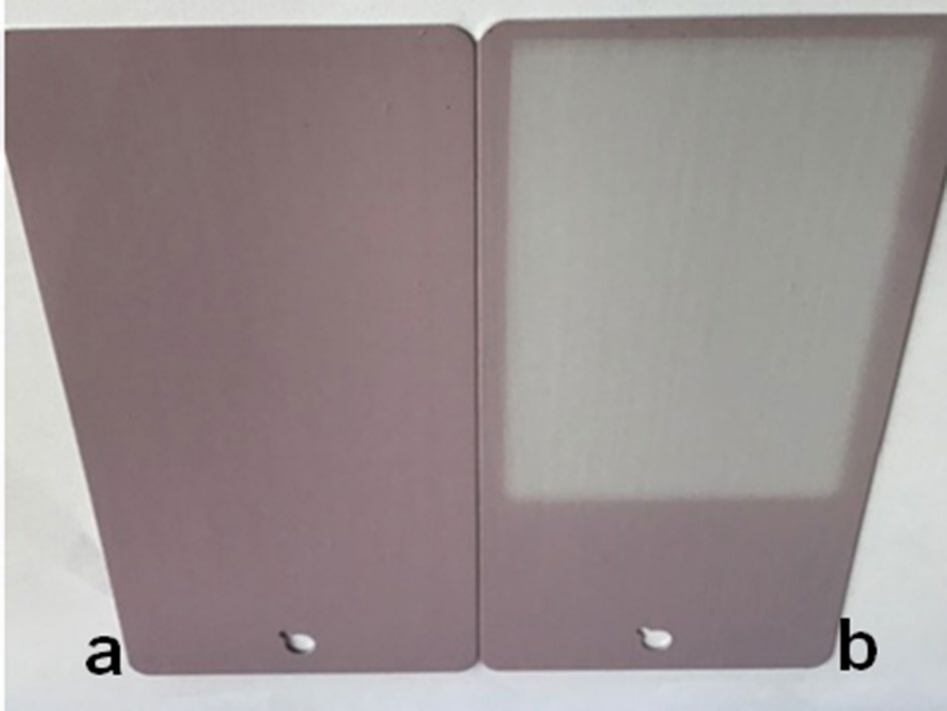
Colour fastness of the dye **I**-APU film under UV exposure (**a**) initial film; (**b**) after 414 h.

According to the DIN-EN-13300, the film falls into the wet abrasion resistance number 2 class. The dye **I**-WB standard and the dye **I**-WB after 200 wet-scrub cycles reflectance spectra (See Supplementary Information, Fig. S2) reveal also that the colour strength K/S is not influenced too much by this fastness test (Table 1). On the other hand, the colour intensity of the dye **I**-APU film under UV radiation obviously decreases with the increasing of the time exposure (Fig. 1), according to the colour parameters and to the colour differences (Table 2)^21^.

**Table 2:** The colour properties of the dye **I**-WB under UV exposure.

Film	L*	a*	b*	C*	h*	ΔE*	ΔC*	Δh*
dye I-APU standard	70.0 ± 0.2	9.5 ± 0.2	5.4 ± 0.1	10.9 ± 0.2	5.8 ± 0.05	–	–	–
dye I-APU after 16 h	72.2 ± 0.1	7.7 ± 0.1	− 3.9 ± 0.1	8.6 ± 0.2	5.8 ± 0.05	9.7 ± 0.2	2.3 ± 0.2	9.2 ± 0.1
dye I-APU after 44 h	77.3 ± 0.2	5.0 ± 0.1	− 3.0 ± 0.2	6.2 ± 0.2	5.8 ± 0.05	12 ± 0.2	4.7 ± 0.2	8.3 ± 0.1
dye I-APU after 86 h	78.0 ± 0.2	4.8 ± 0.2	− 3.1 ± 0.1	5.7 ± 0.2	5.7 ± 0.05	12.3 ± 0.2	5.2 ± 0.2	8.0 ± 0.1
dye I-APU after 209 h	79.8 ± 0.2	2.1 ± 0.2	− 2.6 ± 0.1	3.4 ± 0.2	5.4 ± 0.05	14.7 ± 0.2	7.5 ± 0.1	7.9 ± 0.1
dye I-APU after 302 h	80.5 ± 0.1	1.5 ± 0.1	− 2.0 ± 0.2	2.5 ± 0.2	5.4 ± 0.05	15.1 ± 0.2	8.4 ± 0.2	6.9 ± 0.1
dye I-APU after 414 h	81.9 ± 0.2	0.6 ± 0.1	− 1.6 ± 0.2	1.7 ± 0.2	5.1 ± 0.05	16 ± 0.2	9.2 ± 0.2	5.5 ± 0.1

**Figure 2:**
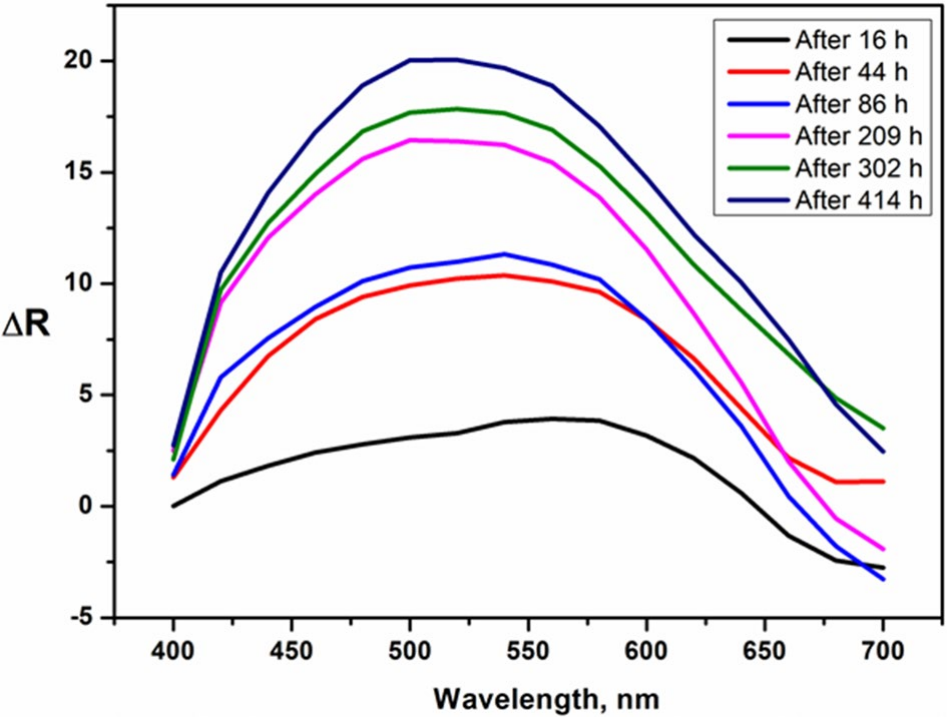
The difference in the reflectance spectra of the dye **I**-APU standard and the dye **I**-APU under UV radiation for some periods of time.

The difference in the reflectance spectra of the dye **I**-APU standard and the dye **I**-APU under UV radiation for some periods of time (Fig. 2) reveals that the colour properties are greatly influenced by this fastness test, because it affects the double bond structures. It may also be specified, that the maxima of these differences appear at lower wavelength as the time of UV exposure is greater^36^. Although the UV tests show that the dye **I** does not resist to UV exposure, the quality of the film is untainted, the degree of gloss even after 414 h of UV exposure remaining the same.

### Adsorption of the new dye *I* studies

The adsorption study from present paper is intended to a concrete, practical destination and therefore the studied parameters are those that control the industrial processes. In real working conditions, the wastewaters generated by industrial plants, even resulted from different processes, are usually collected in equalization (homogenization) tanks before treatment. Therefore, the adsorption of dyes from technological waters, resulted from technologic equipment, occurs at relatively constant pH values. For this reason, the influence of pH on the dye adsorption process was not take into account.

#### Effect of contact time

**Figure 3:**
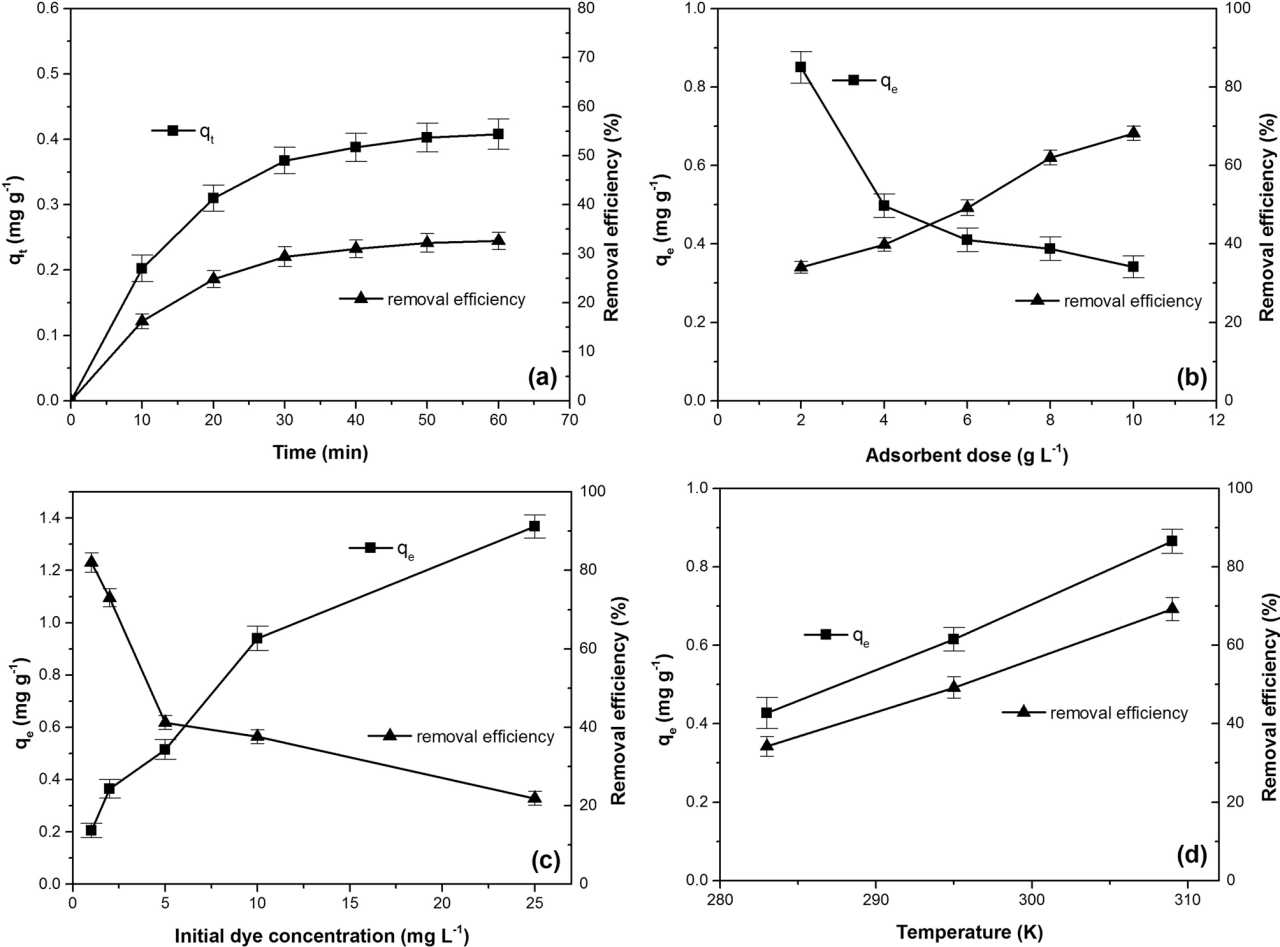
Effect of (**a**) time, (**b**) adsorbent dose, (**c**) initial dye concentration, (**d**) temperature on dye **I** adsorption onto PAC. (Adsorption conditions: (**a**): initial dye concentration: 5 mg L^−1^; adsorbent dosage: 4 g L^−1^; temperature: 295 K, (**b**): initial dye concentration: 5 mg L^−1^; contact time: 60 min; temperature: 295 K, (**c**): contact time: 60 min; adsorbent dosage: 4 g L^−1^; temperature: 295 K, (**d**): initial dye concentration: 5 mg L^−1^; contact time: 60 min; adsorbent dosage: 6 g L^−1^).

Figure 3a reveals that the adsorption capacity and the dye removal efficiency increase with the increase of the contact time. These facts may be explained by a large number of surface sites available at the beginning of the process. As time increases further, the number of the available sites decreases (more surface sites are occupied) leading to the slow increase of adsorption parameters. In this stage, the dye diffusion may occur in adsorbent pores as well^26,37^. The equilibrium was reached after 50–60 min when the majority of adsorption sites are covered by the dye **I** molecules.

#### Effect of adsorbent dose

The adsorbent amount is an important parameter that influences the adsorption process. Increasing the dose of adsorbent has a positive effect on the efficiency of the dye removal (Fig. 3b). A large number of dye **I** molecules are adsorbed due to the increase of the adsorption surface and the availability of the active adsorption sites number^30,37–39^. As the process progresses, two phenomena can occur that lead to a decrease of adsorption capacity: (i) the adsorption sites remain unsaturated, whereas, the sites number available for adsorption increases and (ii) aggregation or agglomeration of adsorbent particles can occur, reducing the available surface area and increasing the diffusion path length^30,38,39^.

#### Effect of initial dye concentration

The initial dye concentration is an important factor that affect the adsorption process influencing the adsorption capacity of the adsorbent and the dye removal efficiency (Fig. 3c). When the initial concentration of the dye **I** increases, an important mass gradient occurs between the solution and the adsorbent material and the driving force increases, which exceeds all the dye mass transfer resistance. In consequence, the adsorption capacity increases and, furthermore, the number of collisions between the dye molecule and the adsorbent particle increases favouring the adsorption process^26,27,37,39,40^. At dye **I** lower concentration, the available active sites on the surface of the adsorbent is higher than the number of the dye particles and the adsorption is more efficient, but when the concentration is increased, the available sites of adsorption become fewer and are quickly occupied. More dye molecules are accumulated on the adsorbent surface and the adsorption sites become saturated, causing the decreasing of the removal efficiency^41,42^.

#### Effect of temperature

Increasing the temperature of the adsorption process (Fig. 3d) leads to an increase in the adsorption capacity and in the dye’s removal efficiency, indicating that the adsorption process is endothermic in nature^27,43^. The solution viscosity is reduced and the dye **I** molecules mobility is also favoured by the increase of the temperature, therefore in the external boundary layer and in the internal adsorbent pores, the dye diffusion is intensified^27,40^.

One of the objectives of the adsorption studies is the determination of the optimum conditions for removal of the traces of dyes from aqueous solutions. According to Fig. 3, the optimal values ​​for the parameters that influence the process were contact time 60 min, adsorbent dose 10 (g L^−1^) and temperature 309 K. At different initial concentrations of the dye **I**, the removal efficiency was determined in optimal conditions (Supplementary material, Fig. S3). The efficiency over 90% was obtained for dye **I** concentrations range 1–10 (mg L^−1^).

#### Equilibrium and kinetic modelling

Adsorption isotherms are fundamental in describing the interactive behaviour between the adsorbate and the adsorbent, and provide important information about the adsorption mechanism, the surface properties and the affinities of the adsorbent under the system condition. The Langmuir, Freundlich and Sips isotherms were employed to investigate the adsorption process. Langmuir isotherm assumes the monolayer adsorption, on a homogeneous surface, without interaction between the adsorbed molecules. Freundlich isotherm assumes multilayer adsorption on the adsorbent heterogeneous surface^44–46^. The Sips isotherm is a combination of the Langmuir and Freundlich isotherms, used to characterize heterogeneous adsorption systems and to avoid limiting the increasing adsorbent concentration associated with the Freundlich model^47^.

Figure S4 in the Supplementary Information illustrates the Langmuir, Freundlich and Sips adsorption isotherms for the dye **I** adsorption on PAC. Isotherms constants, calculated from the slope and intercept of the plots are presented in Table 3. The values of determination coefficient (R^2^), sum of square error (SSE), chi-square (χ^2^) and average relative error (ARE) suggest that the adsorption process is better described by the Sips isotherm. This means that the adsorption process follows the Freundlich model (diffused adsorption) at low dye concentration and the Langmuir model (monomolecular adsorption with a saturation value) at high dye concentrations^47^.

**Table 3:** Adsorption isotherms and kinetic models constants and the corresponding error functions.

Isotherm model	Parameters	Value	Kinetic model	Parameters	Value
Langmuir	K_L_ (L mg^−1^)	0.331 ± 0.139	Pseudo-first order	k_1_ (min^−1^)	0.066 ± 0.001
	q_max_ (mg g^−1^)	1.508 ± 0.196		q_e_,_calc_ (mg g^−1^)	0.419 ± 0.002
	R^2^	0.9715		R^2^	0.9996
	χ^2^	2.62 × 10^−1^		χ^2^	1.17 × 10^−4^
	SSE	4.52 × 10^−2^		SSE	3.73 × 10^−5^
	ARE (%)	43.95		ARE (%)	17.30
Freundlich	*K*_f_ (mg g^−1^)	0.458 ± 0.017	Pseudo-second order	k_2_ (g mg^−1^ min^−1^)	0.122 ± 0.018
	1/n	0.372 ± 0.115		q_e_,_calc_ (mg g^−1^)	0.535 ± 0.017
	R^2^	0.9968		R^2^	0.9971
	χ^2^	0.75 × 10^−2^		χ^2^	1.15 × 10^−3^
	SSE	0.2 × 10^−2^		SSE	3.46 × 10^−4^
	ARE (%)	4.25		ARE (%)	18.67
Sips	Q_sat_	3.921 ± 1.081		q_e_,_exp_ (mg g^−1^)	0.407 ± 0.021
	K_F_	0.131 ± 0.041			
	1/n	2.109 ± 0.037			
	R^2^	0.9993			
	χ^2^	0.11 × 10^−2^			
	SSE	0.57 × 10^−3^			
	ARE (%)	2.39			

The kinetic study provides useful information (adsorption mechanism and efficiency) for selecting optimum operating conditions for the industrial scale processes^29,48,49^. The pseudo-first-order and pseudo-second-order models were used to fit the experimental data (See Supplementary Information, Fig. S5). Kinetic parameters models and the error functions values, summarized in Table 3, and the calculated equilibrium adsorption capacity (in good agreement with the experimental values), indicate that the pseudo-first order kinetic model best describes the adsorption process.

#### Thermodynamic parameters

The values for thermodynamic parameters, standard Gibbs free energy change (ΔG^0^), standard enthalpy change (ΔH^0^) and standard entropy change (ΔS^0^), presented in Table S1 in the Supplementary Information file and calculated from the slope and the intercept of ln K_L_ versus 1/T plot (See Supplementary Information, Fig. S6) indicate that the dye **I** adsorption is a spontaneous, favourable and endothermic process. The positive value of standard entropy change (ΔS^0^) shows the affinity of adsorbent for dye **I** and suggests the increased disorder and randomness (the degrees of freedom of the adsorbed species) at the solid solution interface^30,37,48^. The value of ΔH^0^ lower than 40 (kJ mol^−1^) indicates that the physical adsorption is involved in the process ^45,49^, moreover if ΔH^0^ is lower than 20 (kJ mol^−1^) van der Waals interactions are implied, with an important contribution, in the physisorption^50^. When standard Gibbs free energy change (ΔG^0^) range from − 20 to 0 (kJ mol^−1^), physisorption is involved in process and when ΔG^0^ is between − 80 and − 200 (kJ mol^−1^), chemical adsorption occurs in process. The values of this parameter suggest that the adsorption is physisorption enhanced by the chemical effect^51^.

#### Colour analysis

The colour loss of the dye **I** solution with a concentration of 5 (mg L^−1^) at different adsorption conditions (Table 4 and Supplementary Information, Figure S7) reveals that after 30 min the lightness L* increases with 1.69 units, but after another 30 min the increase is only of 1.25 units. At the same time, the colour parameter a* diminishes its value with 0.82 units and the parameter b* increases its value with 2.22 units after 30 min. After 60 min, the parameter a* diminishes its value with 0.22 units, while the parameter b* increases its value with 0.86 units. In the absorbance spectra (See Supplementary Information, Fig. S7a) the maximum reduces its intensity with the adsorption time. This may be explained taking into account that, while the adsorption process progresses, adsorption centres are fewer. When the adsorbent dose increases twice, with the same amount of 4 (g L^−1^), maintaining the same temperature and the same contact time, the colour parameters change almost with the same differences among them, but the absorbance maximum decreases with the adsorbent amount (See Supplementary Information, Fig. S7b). Working with an adsorbent dose of 6 (g L^−1^) at 60 min contact time, the adsorption process is more efficient at higher temperature. The colour parameters L* and b* increase while a* is reduced and therefore, the dye solution becomes lighter and the absorbance maximum decreases (See Supplementary Information, Fig. S7c). The colour analysis confirms the results obtained at adsorption, as presented above, and sustains that the active carbon powder used as adsorbent is efficient for removal the dye **I** from water solutions.

**Table 4:** Colour parameters at different adsorption conditions of the dye solution.

Adsorption conditions		Colour parameters		
Adsorbent dose 4 (g L^−1^), temperature 295 K	Contact time (min)	L*	a*	b*
	0	84.35 ± 0.04	1.65 ± 0.01	− 6.73 ± 0.01
	30	86.04 ± 0.01	0.83 ± 0.01	− 4.51 ± 0.01
	60	87.29 ± 0.02	0.61 ± 0.01	− 3.65 ± 0.01
Contact time 60 min, temperature 295 K	Adsorbent dose (g L^−1^)	L*	a*	b*
	2	85.44 ± 0.01	0.87 ± 0.01	− 3.71 ± 0.01
	6	86.65 ± 0.01	0.52 ± 0.02	− 2.85 ± 0.01
	10	88.85 ± 0.01	0.16 ± 0.01	− 1.92 ± 0.01
Contact time 60 min, adsorbent dose 4 (g L^−1^)	Temperature (K)	L*	a*	b*
	283	85.54 ± 0.02	0.87 ± 0.01	− 4.87 ± 0.01
	295	86.65 ± 0.01	0.52 ± 0.02	− 2.85 ± 0.01
	309	87.45 ± 0.01	0.31 ± 0.01	− 2.04 ± 0.01

## Materials and methods

**Figure 4:**
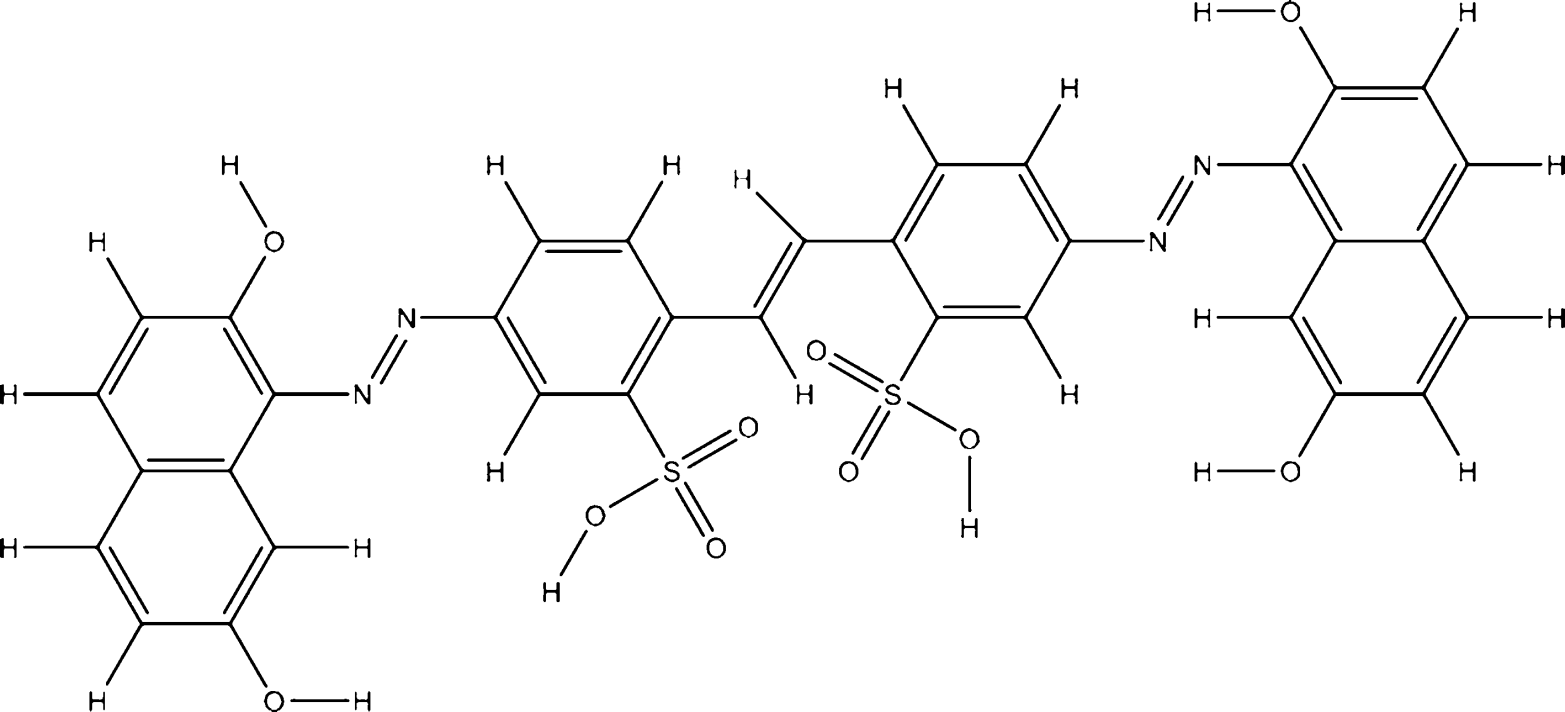
Structure of the dye **I** (reproduced and adapted from reference number^31^, with permission from Elsevier).

A new direct symmetrical azo-stilbene dye namely dye **I** (Fig. 4) for acrylic resins was synthetized using 4,4′-diaminostilbene-2,2′-disulfonic acid, as middle component and 2,7-dihydroxynaphthalene, as coupling component. Its structure, the obtaining method and the main physico-chemical properties are presented in a previous paper^31^.

### Fastness properties

The chemicals employed in this study are the new dye **I**, a water-based single component acrylic resin (WB) and a solvent-based two components acrylo-polyurethanic resin (APU), both compositions containing 65% titanium dioxide. The materials used for films deposition were a PCV foil and an aluminium plate, respectively. All materials were provided by AZUR S.A.

The colour fastness measurements of the applied dye **I** were performed on two types of films: a water-based acrylic resin (dye **I**-WB) and a solvent-based acrylo-polyurethanic resin (dye **I**-APU).

The colour CIEL*a*b* parameters of the both films were determined using a MINOLTA CM 3220d spectrophotometer in the following conditions: the CIE D65 illuminant (natural day light) and the standard 10° observer function. All colour data were expressed by L*, a*, b* coordinates*,* where L* corresponds to lightness; a* corresponds to the transition from green (− a*) to red (+ a*); and b* corresponds to the transition from blue (− b*) to yellow (+ b*).

The wet-scrub resistance was determined with a friction test apparatus, according to EN ISO 11998/2001. The colour differences between the original film (dye **I**-WB) applied on a PCV foil and after 200 wet-scrub cycles one were determined.

The abrasion resistance was determined according to DIN-EN-13300.

For UV fastness tests, the dye **I**-APU applied on an aluminium plate was exposed under UV light for 414 h, periodically monitoring the colour parameters. The degree of gloss was also tested for different periods of time.

The UV film tests were performed with a QUV Accelerated Weathering Tester, model spray from Q-Lab according to SR EN ISO 16474-3-2013.

The film degree of gloss was determined with a Rhopoint Instruments 20/60/85° Gloss-Haze-Doi/RIQ Meter.

### Adsorption studies

Powered activated carbon (PAC) provided by Merck was used in the experiments. The batch adsorption studies were conducted at constant mixing intensity (provided by a M.T.A. 609/A shaker) in Erlenmeyer flasks (150 mL), where the dye **I** solutions were agitated with PAC. The dye concentrations were measured by a UV–VIS spectrophotometer Specord 200 PLUS at 560 nm wavelength. Three independent replicates were realized for each adsorption experiment.

Non-linear forms of Langmuir, Freundlich and Sips isotherms and pseudo-first order and pseudo-second order kinetic models were analyzed according to the equations described in literature^29,30,38,51–53^. In order to establish the best-fitting model for the dye adsorption, the values of determination coefficient (R^2^), sum of square error (SSE), chi-square (χ^2^) and average relative error (ARE) were taking into consideration^53^.

The thermodynamic parameters (standard Gibbs free energy change, standard enthalpy change and standard entropy change) were computed, adopting the equations described in previous adsorption studies^37,51,54^, using the obtained experimental data at temperatures of 283, 295 and 309 K.

The colour analysis of the adsorption samples was conducted using a Cary-Varian 300 Bio UV–VIS colourimeter with integrating sphere, using a Spectralon standard and the illuminant D65, and the standard 10° observer function.

## Conclusions

In the film field domain, the wet-scrub and the UV are reference tests. After the new dye **I** was submitted to these tests, the colour of the dye **I**-WB film under wet-scrub test resists with good properties after 200 cycles, and the film falls into the wet abrasion resistance number 2 class. Under UV exposure, the coloured dye **I**-APU film do not resist, due to the double bonds structures damages. The maxima of the reflectance spectra are inversely proportional to the UV time exposure. The degree of gloss remains the same, revealing that the film quality does not change.

The adsorption studies showed that the Sips isotherm and the pseudo-first order kinetic model describe the process. Thermodynamic parameters revealed that the adsorption is a spontaneous, favourable and endothermic process, moreover, is a physisorption (with van der Waals interactions implied) enhanced by the chemical effect. The colour analysis confirms the results obtained at adsorption, and sustains that the PAC adsorbent is efficient for removal of the dye **I** from water solutions.

## Supplementary Information

Supplementary Information
