# Supplementary material for: Aspects regarding colour fastness and adsorption studies of a new azo-stilbene dye for acrylic resins
Source: Sci Rep. 2021 Mar 15;11:5889. doi: 10.1038/s41598-021-85452-7 (PMC7971012; doi:10.1038/s41598-021-85452-7)
Supplement: Supplementary file 1 — Supplementary Information [file 41598_2021_85452_MOESM1_ESM.docx]

**Aspects regarding colour fastness and adsorption studies of a new azo-stilbene dye for acrylic resins**

Simona Popa^1^, Maria Elena Radulescu-Grad ^2^*, Alina Perdivara^3^, Giannin Mosoarca^1^*

^1^ Politehnica University of Timisoara, Faculty of Industrial Chemistry and Environmental Engineering,

V. Parvan Bd. No. 6, 300223, Timisoara, Romania

^2^“Coriolan Dragulescu" Institute of Chemistry, Romanian Academy, Mihai Viteazul Bd. No. 24, 300223, Timisoara, Romania

^3^AZUR S.A., Constructorilor Bd., No. 1-3, 300571Timisoara, Romania

simona.popa@upt.ro; *corresponding author: m.radulescugrad@gmail.com; alina.perdivara@azur.ro *corresponding author: giannin.mosoarca@upt.ro

**Results and Discussion**

**Fastness properties.**

The colour of the dye **I**-WB film under the wet-scrub test slightly changed as can be seen in Fig. S1.


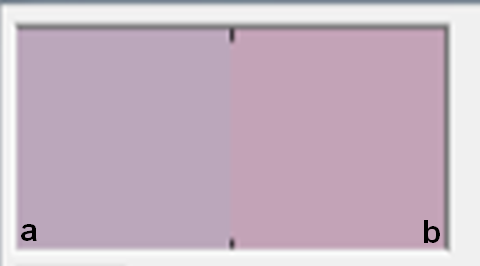


**Figure S1.** Colour fastness of the new dye **I**-WB film at wet-scrub test:

a - initial film; b - after 200 cycles

The reflectance spectra of the dye **I**-WB standard and the dye **I**-WB after 200 wet-scrub cycles reveal that they are similar (Fig. S2).


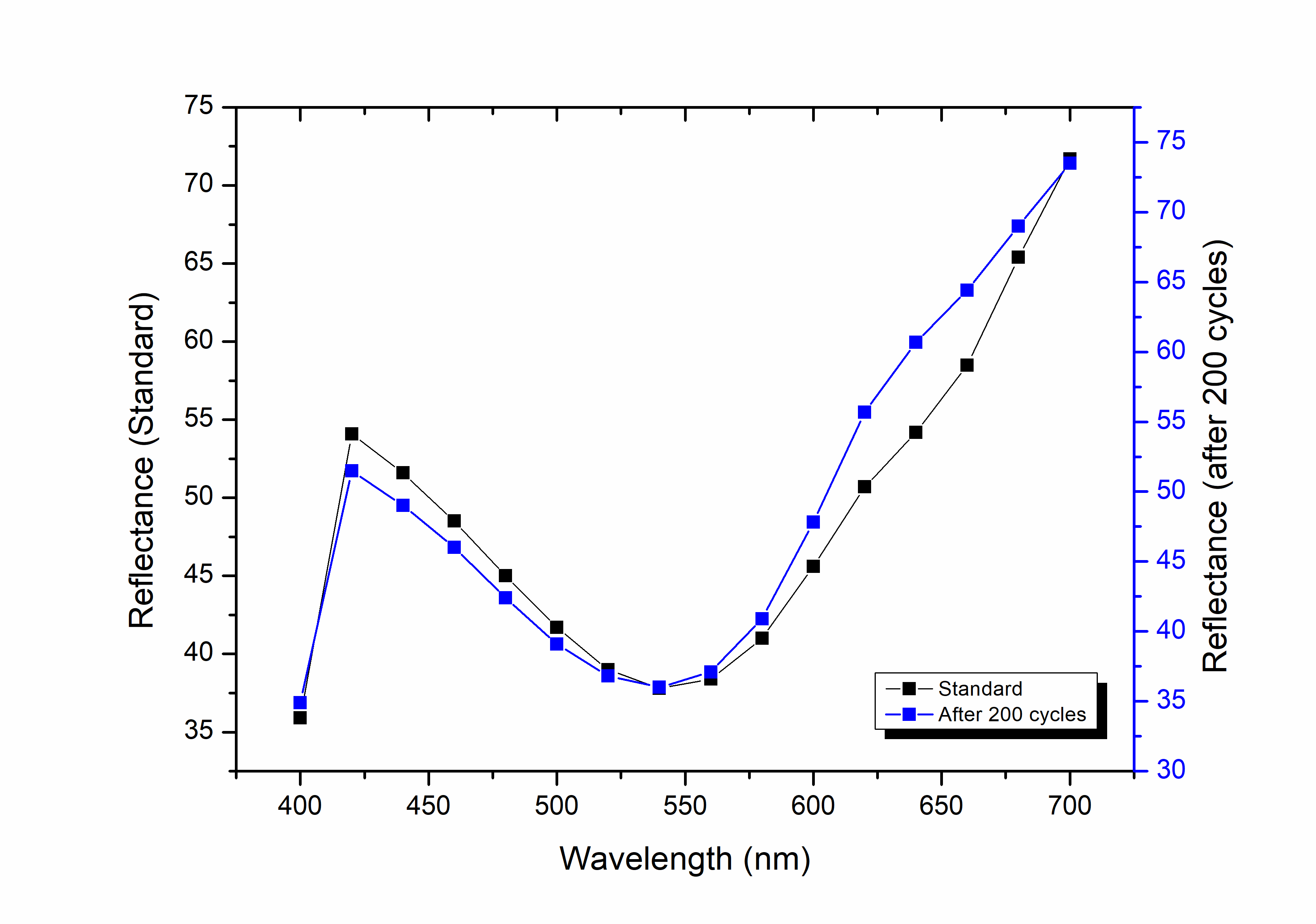


**Figure S2.** The dye **I**-WB standard and the dye **I**-WB after 200 wet-scrub cycles reflectance spectra

**Adsorption of the new dye I studies.**

Removal efficiency at different initial dye concentration, in optimum conditions is presented in Fig.S3.

**Figure S3.** Removal efficiency at different initial dye concentration, in optimum conditions (contact time: 60 minutes, adsorbent dose: 10 g L^-1^ and temperature: 309 K)

Figure S4 illustrate the Langmuir, Freundlich and Sips adsorption isotherms for the dye adsorption on PAC.

**Figure S4.** Langmuir, Freundlich and Sips isotherms for dye adsorption on PAC

(contact time: 60 min; adsorbent dosage: 4 g L^-1^; temperature: 295 K)

The pseudo-first-order and pseudo-second-order models were used to fit the experimental data (Fig. S5).

**Figure S5.** Pseudo-first order and pseudo-second order kinetic models for dye adsorption on PAC (initial dye concentration: 5 mg L^-1^; adsorbent dosage: 4 g L^-1^; temperature: 295 K)

The values for thermodynamic parameters are presented in Table S1. They were calculated from the slope and the intercept of ln K_L_ versus 1/T plot (Fig. S6) and indicate that the dye adsorption is a spontaneous, favourable and endothermic process.

| **ΔG^0^ (kJ mol^-1^)** | | | **ΔH^0^ (kJ mol^-1^)** | **ΔS^0^ (J mol^-1^)** |
| --- | --- | --- | --- | --- |
| 283 K | 295 K | 309 K |  |  |
| -28.19 | -30.49 | -32.84 | 2.69 | 21.54 |

**Table S1.** The values of the standard Gibbs free energy change (ΔGº), standard enthalpy change (ΔHº) and standard entropy change (ΔSº)

**Figure S6.** Plot of ln K_L_ vs. 1/T for dye adsorption on PAC

**Colour Analysis.** The decrease of the absorbance maximum of the dye solutions with a concentration of 5 (mg L^-1^) at different adsorption conditions are presented in Figure S7.


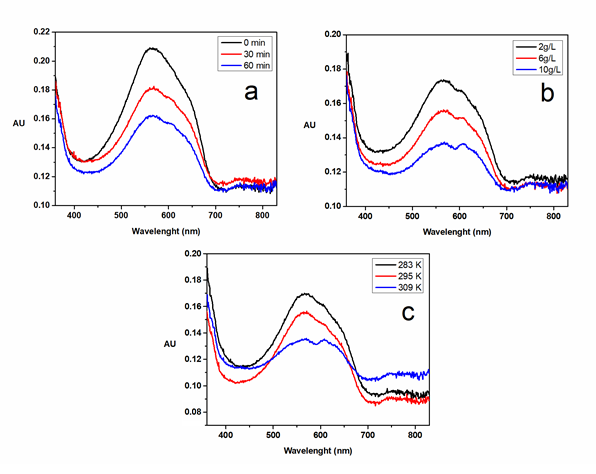


**Figure S7.** The absorbance spectra of the adsorption processes: **(a)** different contact times, **(b)** different adsorbent doses, **(c)** different temperatures
